# Original Synthesis of Fluorenyl Alcohol Derivatives by Reductive Dehalogenation Initiated by TDAE

**DOI:** 10.3390/molecules21101408

**Published:** 2016-10-24

**Authors:** Alain Gamal Giuglio-Tonolo, Thierry Terme, Patrice Vanelle

**Affiliations:** Aix-Marseille Université, CNRS, Institut de Chimie Radicalaire ICR, UMR 7273, Laboratoire de Pharmaco-Chimie Radicalaire, Marseille 13385, France; gamal.giuglio-tonolo@univ-amu.fr (A.G.G.-T.); thierry.terme@univ-amu.fr (T.T.)

**Keywords:** tetrakis(dimethylamino)ethylene, reductive dehalogenation, single electron transfer

## Abstract

We report here a novel and easy-to-handle reductive dehalogenation of 9-bromofluorene in the presence of arylaldehydes and dicarbonyl derivatives to give the corresponding fluorenyl alcohol derivatives and Darzens epoxides as by-products in tetrakis(dimethylamino)ethylene (TDAE) reaction conditions. The reaction is believed to proceed via two successive single electron transfers to generate the fluorenyl anion which was able to react with different electrophiles. A mechanistic study was conducted to understand the formation of the epoxide derivatives.

## 1. Introduction

Fluorenyl derivatives have attracted interest from synthetic organic chemists due to their notable biological and pharmaceutical properties and applications. The literature reports that fluorenone Schiff base derivatives have a potential application and biological activity in therapeutic areas, such as antifungal agents [1,2]. The fluorene skeleton is found in numerous natural products. Dendroflorin and structurally-related nobilone displayed higher antioxidant activity than vitamin C in the ORAC assay [3]. Gramniphenols were isolated from the whole plant of *Arundina gramnifolia* by Yang and coworkers in 2012 and displayed anti-HIV-1 activity with therapeutic index values above 100:1 [4]. Many dermatological and photostable cosmetic compositions consist of lumefantrine, a 9-fluorenylidene derivative [5]. The fluorenyl core can be found in indecainide (Decabid^®^), which is a class Ic anti-arrhythmic agent [6].

**Figure 1:**
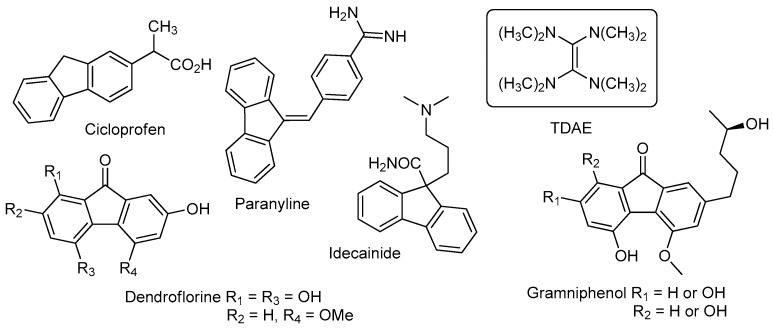
Structures of biological active molecules and TDAE.

In terms of non-steroidal anti-inflammatory agents (NSAIDs), this scaffold bearing an alkanoïc acid moiety gives cicloprofen. Paranyline is used in dispersible formulations of anti-inflammatory agents [7]. However, although these drugs are very effective and abundantly prescribed, they have revealed some gastric or intestinal adverse effect [8]. The representatives containing the fluorenyl scaffold cited above are shown in Figure 1.

Since 2003, our laboratory has been developing original synthetic methods based on neutral, ground-state organic reducing agents [9,10,11,12,13,14], aimed at preparing new, potentially bioactive compounds [9]. Tetrakis(dimethylamino)ethylene (TDAE), as shown in Figure 1 [15,16,17], is a powerful tool in modern organic chemistry because it allows reduction under mild conditions. Reduction can occur without the use of organometallics or reactive metals. TDAE shows a single, two-electron oxidation peak at −0.62 V vs. SCE in diméthylformamide (DMF) which means it is about as reducing as zinc metal [18,19,20]. TDAE can react with activated alkyl halides, such as CF_3_I, CF_2_Br_2_, and activated benzyl halides, to produce the corresponding carbanions [21,22,23,24,25,26,27,28,29,30]. We report herein a valuable synthetic method to generate a 9-bromofluorenyl anion from fluorenyl bromide **1** and on its reaction with a series of carbonyl electrophiles affording the corresponding alcohol derivatives.

## 2. Results and Discussion

**Scheme 1:**
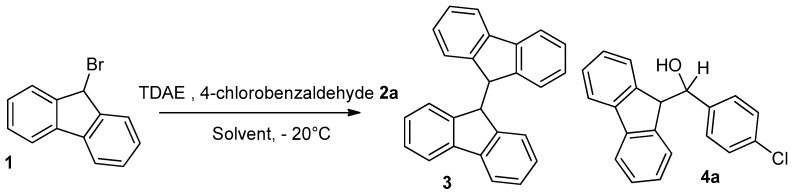
Optimization of the reaction of fluorenyl bromide **1** with 4-chlorobenzaldehyde **2a** using TDAE.

It was anticipated that fluorenyl bromide **1** could be a good electron-acceptor, because of its large π-conjugated system. Initially, the reaction of 9-bromofluorene **1** and 4-chlorobenzaldehyde **2a** was investigated as the model system (Scheme 1).

We performed the reactions using various aprotic polar solvents. The influence of the quantity of aldehyde **2a** and TDAE was also studied. The results are summarized in Table 1.

A first attempt (entry 1) was carried out with 2 equiv. of **2a** and 1 equiv. of RBr **1** to give 29% of the corresponding alcohol **4a**. TDAE was added at −20 °C to maximize the charge transfer complex formation. Unfortunately, ^1^H NMR spectra of the crude product showed a signal at δ 4.85 associated to the homo-coupling adduct of 9-bromofluorene (44%). To reduce the extent of dimer formation, 3 equiv. of **2a** was employed (entry 2), giving 50% of **4a** and the dimer was isolated with 8% yield (entry 2). The conversion to the 9,9′-bifluorenyl was not reduced by increasing the excess of aldehyde. The optimized protocol of 9-bromofluorene **1** was defined with 3 equiv. of aldehyde **2a**, 1 equiv. of TDAE in DMF, for 1 h at −20 °C followed by 2 h at room temperature. The reactions led to the corresponding alcohol **4a** with 56% yield (Table 1, entry 6). The 9-Bromofluorene was found to be a good substrate in such TDAE-mediated carbon-carbon coupling reactions. This methodology was then applied to other electrophiles. The TDAE-initiated reaction of **1** with various electrophiles was investigated according to the optimal procedure cited above and the results are summarized in Table 2. The expected corresponding alcohols **4a**, **4b**, **4e**, **4f**, **4i** were isolated in moderate to good yields, from 41% to 75% with aldehydes bearing an electron-withdrawing group.

For 4-nitrobenzaldehyde **2h**, no alcohol product was isolated. Its strong electrophilic character, has already led to unexpected reactivity in these reaction conditions, as cited in our previous work [11]. With methylbenzaldehyde **2g** and 4-methoxybenzaldehyde **2d**, the conversions were lower with, respectively, 24% and 34% yields. This may be explained by their weak electrophilic character compared to the other aldehydes. Competitive epoxide formation was observed, which was obtained in low yield (from 6% to 30%, Table 2, entries 1–9). Subsequently, the methodology was generalized to heteroaromatic aldehydes (entries 10–12). When pyridine-4-carbaldehyde **2k** was employed, 42% of the corresponding alcohol **4k** was obtained. With pyridine-3-carbaldehyde **2l**, the yield reported was lower (19%). The scope of this original reactivity was extended to pyruvates, ethyl glyoxylate, methyl 3,3,3-trifluoropyruvate, and diethylketomalonate (entries 13–16). The best result was achieved with diethylketomalonate affording the corresponding fluorenyl alcohol **4p** in 70% yield (entry 16). This can, no doubt, be attributed to the stronger electrophilic character of its carbonyl function. It should be noted that we never observed the reaction of the fluorenyl anion with ester moiety. The poor yield (19%) observed with ethyl glyoxylate **2o** could be due to a polymerization reaction of the aldehyde. With propriophenone **2q**, only starting materials were recovered (entry 17), which could be attributed to the acidity of the protons adjacent to the carbonyl function.

In order to extend the substrate scope of TDAE-initiated reactions, an attempt had been run with 1,1′-bromobiphenylmethane, but no alcohol adduct was formed. The corresponding dimer product was obtained with 74% yield. This difference in reactivity can be attributed to the better stabilization of the corresponding 9-fluorenyl aromatic anion.

**Scheme 2:**
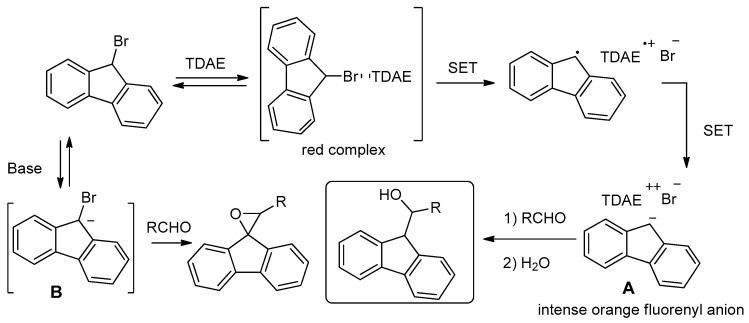
Proposed mechanism.

The formation of the alcohol product can be conceived to proceed via two successive single electron transfers (Scheme 2). The first reduction leads to the cleavage of the C-Br bond to form the 9-fluorenyl radical and the bromide anion. A subsequent SET (single monoelectronic transfer) gave the fluorenyl anion A, which is aromatic and has an intense orange color, from the corresponding radical. An alcoholate was successively obtained by the nucleophilic addition of the fluorenyl anion A on the aldehyde which gave the corresponding alcohol after hydrolysis. As cited above, 9-bromofluorene 1 can react under our conditions with aromatic aldehydes, yielding epoxy products. Our hypothesis is that 9-bromofluorenyl anion B could be formed after deprotonation of 1 by the above alcoholate. The reaction of B with the aldehyde gave the epoxide derivatives via Darzen reaction. Our hypothesis is supported by literature data [31].

**Scheme 3:**
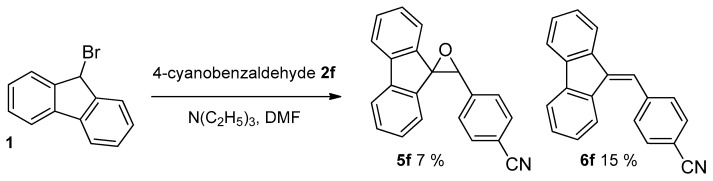
Reactivity of 9-bromofluorene and 4-cyanobenzaldehyde in the presence of trimethylamine.

When 9-bromofluorene **1** was treated with 3 equiv. of triethylamine in the presence of 4-cyanobenzaldehyde **2f** under classical TDAE conditions, the corresponding Darzens condensation product **5f** was afforded in low yields (7%) (Scheme 3).

Another compound had been observed by ^1^H-NMR corresponding to 4-((9*H*-fluoren-9-ylidene)methyl)benzonitrile **6f** with 15% yield. However, another multistep pathway involving a carbene-like intermediate cannot be excluded. To verify this hypothesis, we examined the reaction of **1** in the presence of cyclohexene [32]. The ^1^H-NMR spectrum of the crude mixture showed no signal attributable to a cyclopropane adduct that could formed by carbene insertion to the cyclohexene double bond: only peaks due to dimer **3** were found. These findings indicate that the epoxide derivative could be formed through the 9-bromofluorenyl anion sequence.

## 3. Materials and Methods

### 3.1. General Information

Melting points were determined on a Büchi melting point B-540 apparatus (BÜCHI Labortechnik AG, Flawil, Switzerland) and are uncorrected. Element analyses were performed on a Thermo Finnigan EA1112 (San Jose, CA, USA) at the spectropole of the Aix-Marseille University. Both ^1^H- and ^13^C-NMR spectra were determined on a Bruker Avance 250 spectrometer (Wissembourg, France) at the Service de RMN de la Faculté de Pharmacie de Marseille of the Aix-Marseille University and on a Bruker Avance III NanoBay 300 MHz spectrometer at the spectropole of Aix-Marseille University. The ^1^H- and the ^13^C-chemical shifts are reported from CDCl_3_ peaks: ^1^H (7.26 ppm) and ^13^C (77 ppm) or Me_2_SO-*d*6 (39.6 ppm). Multiplicities are represented by the following notations: s, singlet; d, doublet; t, triplet; q, quartet; m, a more complex multiplet, or overlapping multiplets. The following adsorbents were used for column chromatography: silica gel 60 (Merck, particle size 0.063–0.200 mm, 70–230 mesh ASTM, (Merck, Darmstadt, Germany). Thin Layer Chromatography (TLC) was performed on 5 cm × 10 cm aluminum plates coated with silica gel 60 F254 (Merck) in an appropriate solvent.

### 3.2. Typical Procedure

The TDAE (0.14 mL, 0.6 mmol) was slowly added, with a syringe, at −20 °C to a vigorously stirred solution of fluorenyl bromide **1** (150 mg, 0.6 mmol) with the appropriate aldehyde (1.8 mmol, 3 equivalents) and a spatula of sodium sulfate in 4 mL of DMF, under air. A red color was immediately developed with the formation of a fine white precipitate. The mixture was then stirred at −20 °C for 1 h and warmed to room temperature over a period of 2 h. Then 0.5 mL of water was added to quench the reaction. The solution was extracted with dichloromethane (3 × 30 mL), the combined organic layers were washed with brine (3 × 40 mL), and dried over MgSO_4_. The crude product was then obtained after evaporation of the solvent under reduced pressure. Purification by silica gel chromatographic column (dichloromethane: methanol) gave the corresponding fluorenyl derivatives.

### 3.3. Compound Characterizations

*9H,9′H-9,9′-Bifluorene*: **3** (9%); yellow powder; m.p. 216–217 °C (Lit.: 237–239 °C). ^1^H-NMR (200 MHz) (CDCl_3_) 7.66 (d, *J =* 7.51 Hz, 4H); 7.33–7.26 (m, 4H); 7.15–7.07 (m, 4H); 6.97 (d, *J =* 7.34 Hz, 4H); 4.85 (s, 2H). ^13^C-NMR (50 MHz) (CDCl_3_) 144.6 (4CIV); 141.5 (4CIV); 127.3 (4CHar); 126.7 (4CHar); 124.0 (4CHar); 119.6 (4CHar); 49.8 (2CH). Physical and spectroscopic data agree with those reported in the literature [33,34].

*(4-Chlorophenyl)(9H-fluoren-9-yl)methanol*: **4a** (56%); yellow powder; m.p. 142–143 °C (Lit.: 126.5 °C). ^1^H-NMR (200 MHz) (CDCl_3_): 7.70 (d, *J =* 7.5 Hz, 2H); 7.40–7.17 (m, 10H); 5.15 (d, *J =* 5.7 Hz, 1H); 4.37 (d, *J =* 5.7 Hz, 1H); 1.90 (bs, OH). ^13^C-NMR (50 MHz) (CDCl_3_): 143.0 (CIV); 142.9 (2CIV); 141.7 (CIV); 140.2 (2CIV); 128.1 (2CHar); 127.7 (2CHar); 126.7 (2CHar); 125.9 (2CHar); 125.5 (2CHar); 119.8 (2CHar); 75.6 (CH); 56.4 (CH). HRMS (ESI^+^): *m/z* [M + Na]^+^ calculated for C_20_H_15_OClNa^+^: 329.0704 Da; found: 329.0704 Da. Physical data agree with those reported in the literature [35].

*3′-(4-Chlorophenyl)spiro(fluorene-9,2′-oxirane)*: **5a** (14%) yellow powder; m.p. 63–64 °C. ^1^H-NMR (200 MHz) (CDCl_3_) 7.76–7.68 (m, 2H); 7.51–7.29 (m, 8H); 7.02–6.94 (dd, *J =* 1 and 7.6 Hz, 1H); 6.50 (d, *J =* 7.6 Hz, 1H); 4.91 (s, 1H). ^13^C-NMR (50 MHz) (CDCl_3_) 141.6 (CIV); 141.4 (CIV); 140.6 (CIV); 138.4 (CIV); 134.0 (CIV); 133.7 (CIV); 129.4 (CHar); 129.3 (CHar); 128.6 (2CHar); 128.3 (2CHar); 127.6 (2CHar); 127.0 (CHar); 123.9 (CHar); 121.6 (CHar); 120.1 (CHar); 67.6 (CIV); 59.1 (CH). HRMS (ESI^+^): *m/z* [M + H]^+^ calculated for C_20_H_14_OCl^+^: 305.0728 Da; found: 305.0728 Da.

*(9H-Fluoren-9-yl)(phenyl)methanol*: **4b** (57%); yellow powder; m.p. 116–117 °C (Lit.: 120–121 °C). ^1^H-NMR (200 MHz) (CDCl_3_) 7.64–7.60 (m, 2H); 7.31–7.24 (m, 7H); 7.16–7.06 (m, 3H); 6.97 (d, *J =* 6.9 Hz, 1H); 5.09 (d, *J =* 6.1 Hz, 1H); 4.40 (d, *J =* 6.1 Hz, 1H); 1.68 (bs, OH). ^13^C-NMR (50 MHz) (CDCl_3_) 143.6 (CIV); 143.3 (CIV); 142.1 (CIV); 141.8 (CIV); 141.7 (CIV); 128.1 (2CHar); 127.8 (CHar); 127.6 (CHar); 126.8 (2CHar); 126.7 (CHar); 126.6 (CHar); 126.6 (CHar);126.2 (CHar); 125.5 (CHar); 119.8 (CHar) ; 119.7 (CHar); 76.3 (CH); 54.7 (CH). HRMS (ESI^+^): *m/z* [M + Na]^+^ calculated for C_20_H_16_ONa^+^: 295.1093 Da; found: 295.1093 Da. Physical and spectroscopic data agree with those reported in the literature [34,36].

*3′-Phenylspiro(fluorene-9,2′-oxirane)*: **5b** (17%); yellow powder; m.p. 119–120 °C (Lit.: 130–131 °C). ^1^H-NMR (200 MHz) (CDCl_3_) 7.76–7.67 (m, 2H); 7.50–7.30 (m, 9H); 6.93 (dt, *J =* 7.6 and 0.9 Hz, 1H); 6.50 (d, *J =* 7.6 Hz, 1H); 4.97 (s,1H). ^13^C-NMR (50 MHz) (CDCl_3_) 141.7 (CIV); 141.5 (CIV); 140.6 (CIV); 138.7 (CIV); 135.2 (CIV); 129.3 (CHar); 129.1 (CHar); 128.3 (2CHar); 128.1 (CHar); 127.6 (CHar); 127.0 (CHar); 126.8 (2CHar); 124.1 (CIV); 121.6 (CHar); 120.2 (CHar); 120.1 (CHar); 67.6 (CIV); 65.7 (CH). HRMS (ESI^+^): *m/z* [M + Na]^+^ calculated for C_20_H_14_ONa^+^: 293.0937 Da; found: 293.0936 Da. Physical and spectroscopic data agree with those reported in the literature [37].

*(4-Bromophenyl)(9H-fluoren-9-yl)methanol*: **4c** (75%); yellow powder; m.p. 129–130 °C. ^1^H-NMR (200 MHz) (CDCl_3_) 7.70 (d, *J =* 7.4 Hz, 2H); 7.43–7.34 (m, 4H); 7.30–7.20 (m, 4H); 7.15 (d, *J =* 8.4 Hz, 2H); 5.15 (d, *J =* 5.5 Hz, 1H); 4.38 (d, *J =* 5.5 Hz, 1H). ^13^C-NMR (50 MHz) (CDCl_3_) 143.0 (2CIV); 142.9 (CIV); 141.8 (CIV); 140.8 (2CIV); 131.1 (2CHar); 128.4 (2CHar); 127.7 (CHar); 126.8 (CHar); 126.7 (CHar); 125.9 (CHar); 125.5 (CHar); 121.5 (CHar); 119.9 (CHar); 119.8 (CHar); 75.7 (CH), 54.6 (CH). HRMS (ESI^+^): *m/z* [M + Na]^+^ calculated for C_20_H_15_OBrNa^+^: 373.0198 Da; found: 373.0198 Da.

*3′-(4-Bromophenyl)spiro(fluorene-9,2′-oxirane)* **5c** (10%) yellow powder; m.p. 122–123 °C. ^1^H-NMR (200 MHz) (CDCl_3_) 7.76–7.69 (m, 2H); 7.57–7.54 (m, 2H); 7.51–7.28 (m, 6H); 7.02–6.96 (m, 1H); 7.51 (d, *J =* 7.7 Hz, 1H); 4.86 (s, 1H). ^13^C-NMR (50 MHz) (CDCl_3_) 141.6 (CIV); 141.3 (CIV); 140.6 (CIV); 138.2 (CIV); 134.2 (CIV); 131.6 (2CHar); 129.5 (CHar); 129.3 (CHar); 128.6 (2CHar); 127.7 (CHar); 127.1 (CHar); 123.9 (CHar); 122.2 (CIV); 121.6 (CHar); 120.2 (2CHar); 67.6 (CIV); 65.1 (CH). HRMS (ESI^+^): *m/z* [M + H]^+^ calculated for C_20_H_14_OBr^+^: 349.0223 Da; found: 349.0150 Da.

*(9H-Fluoren-9-yl)(4-methoxyphenyl)methanol*: **4d** (34%); white powder; m.p. = 115–116 °C (Lit.: 122–123 °C)^3^; ^1^H-NMR (200 MHz) (CDCl_3_) 7.74–7.70 (dd, *J =* 7.6 and 3.3 Hz, 2H); 7.46–7.32 (m, 3H); 7.27–7.15 (m, 4H); 7.03 (d, *J =* 7.6 Hz, 1H); 6.88 (d, *J =* 8.8 Hz, 2H); 4.97 (d, *J =* 6.5 Hz, 1H); 4.38 (d, *J =* 6.5 Hz, 1H); 3.83 (s, 3H) 1.96 (bs, OH). ^13^C-NMR (50 MHz) (CDCl_3_) 159.1 (CIV); 143.9 (CIV); 143.3 (CIV); 141.8 (CIV); 141.6 (CIV); 134.4 (CIV); 128.0 (2CHar); 127.6 (CHar); 127.5 (CHar); 126.6 (CHar); 126.5 (CHar); 126.3 (CHar); 125.7 (CHar); 119.8 (CHar); 119.7 (CHar); 113.5 (2CHar); 76.1 (CH); 55.2 (CH); 54.7 (CH_3_). HRMS (ESI^+^): *m/z* [M + Na]^+^ calculated for C_21_H_18_O_2_Na^+^: 325.1199 Da; found: 325.1200 Da.

*(9H-Fluoren-9-yl)(4-fluorophenyl)methanol*: **4e** (54%); white powder; m.p. 120–121 °C; ^1^H-NMR (200 MHz) (CDCl_3_) 7.70 (d, *J =* 7.4 Hz, 2H); 7.41–7.33 (m, 3H); 7.26–7.14 (m, 5H); 7.01–6.95 (m, 2H); 5.11 (d, *J =* 5.6 Hz, 1H); 4.37 (d, *J =* 5.6 Hz, 1H); 2.01 (bs, OH). ^13^C-NMR (50 MHz) (CDCl_3_) 160.5 (CIV); 143.5 (CIV); 143.2 (CIV); 142.0 (CIV); 141.9 (CIV); 134.8 (CIV); 128.5 (CHar); 128.4 (CHar); 127.7 (2CHar); 126.7 (2CHar); 126.0 (CHar); 125.7 (CHar); 119.9 (CHar) ; 119.8 (CHar); 115.0 (CHar); 114.7 (CHar); 75.9 (CIV); 54.9 (CH). HRMS (ESI^+^): *m/z* [M + Na]^+^ calculated for C_20_H_15_OFNa^+^: 313.0999 Da; found: 313.0999 Da.

*4-((9H-Fluoren-9-yl)(hydroxy)methyl)benzonitrile*: **4f** (41%); white powder; m.p. 169–170 °C; ^1^H-NMR (200 MHz) (CDCl_3_) 7.68 (d, *J =* 7.7 Hz, 2H); 7.52 (d, *J =* 8.2 Hz, 2H); 7.41–7.18 (m, 8H); 5.36 (d, *J =* 4.7 Hz, 1H); 4.42 (d, *J =* 4.7 Hz, 1H); 2.07 (bs, 1H). ^13^C-NMR (50 MHz) (CDCl_3_) 146.7 (CIV); 142.5 (CIV); 142.3 (CIV); 141.8 (2CIV); 131.6 (2CHar); 128.0 (2CHar); 127.3 (2CHar); 127.0 (CHar); 126.8 (CHar); 125.5 (CHar); 125.4 (CHar); 120.1 (CHar); 120.0 (CHar); 118.8 (CIV); 111.3 (CIV); 75.5 (CH), 54.5 (CH). HRMS (ESI^+^): *m/z* [M + H]^+^ calculated for C_21_H_16_NO^+^: 298.1154 Da; found: 298.1154 Da.

*4-(Spiro(fluorene-9,2′-oxiran)-3′-yl)benzonitrile*: **5f** (30%) white powder; m.p. 139–140 °C; ^1^H-NMR (200 MHz) (CDCl_3_) 7.69–7.60 (m, 4H); 7.55–7.52 (m, 2H); 7.47–7.23 (m, 4H); 6.92–6.85 (m, 1H); 6.33 (d, *J =* 7.7 Hz, 1H); 4.86 (s,1H). ^13^C-NMR (50 MHz) (CDCl_3_) 141.1 (CIV); 141.0 (CIV); 140.7 (CIV); 140.6 (CIV); 132.2 (2CHar); 130.5 (CHar); 129.7 (CHar); 129.6 (CHar); 129.1 (CIV); 128.6 (CIV); 127.8 (2CHar); 127.1 (CHar); 123.7 (CHar); 121.6 (CHar); 120.8 (CIV); 120.4 (CHar); 120.3 (CHar); 65.0 (CIV); 60.3 (CH). HRMS (ESI^+^): *m/z* [M + Na]^+^ calculated for C_21_H_13_NONa^+^: 318.0889 Da; found: 318.0889 Da.

*(9H-Fluoren-9-yl)(p-tolyl)methanol* **4g** (24%); yellow powder; m.p. 86–87 °C; ^1^H-NMR (200 MHz) (CDCl_3_) 7.72 (d, *J =* 7.7 Hz, 1H); 7.41–7.04 (m, 11H); 5.02 (d, *J =* 5.0 Hz, 1H); 4.39 (d, *J =* 5.0 Hz, 1H); 2.38 (s, 3H). ^13^C-NMR (50 MHz) (CDCl_3_) 143.7 (CIV); 143.4 (CIV); 141.8 (CIV); 141.7 (CIV); 139.2 (CIV); 137.5 (CIV); 128.9 (2CHar); 127.6 (CHar); 127.5 (CHar); 126.7 (2CHar); 126.6 (2CHar); 126.3 (CHar); 125.6 (CHar); 119.8 (CHar); 119.7 (CHar); 76.2 (CH); 54.7 (CH); 21.2 (CH_3_). HRMS (ESI^+^): *m/z* [M + Na]^+^ calculated for C_21_H_18_ONa^+^: 309.1250 Da; found: 309.1249 Da.

*3′-(4-Nitrophenyl)spiro(fluorene-9,2′-oxirane)* **5h** (17%); yellow powder; m.p. 152–153 °C; ^1^H-NMR (200 MHz) (CDCl_3_) 8.30 (dd, *J =* 8.6 and 2.4 Hz, 2H); 7.77–7.66 (m, 4H); 7.52–7.31 (m, 4H); 6.97–6.94 (m, 1H); 6.45 (d, *J =* 7.7 Hz, 1H); 4.99 (s, 1H). ^13^C-NMR (50 MHz) (CDCl_3_) 147.8 (CIV); 142.4 (CIV); 141.7 (CIV); 140.8 (CIV); 140.6 (CIV); 137.6 (CIV); 129.8 (CHar); 129.6 (CHar); 127.9 (2CHar); 127.8 (CHar); 127.1 (CHar); 123.7 (2CHar); 123.5 (CHar); 121.6 (CHar); 120.5 (CHar); 120.3 (CHar); 68.0 (CIV); 64.9 (CH). HRMS (ESI^+^): *m/z* [M + H]^+^ calculated for C_20_H_14_NO_3_^+^: 315.0895 Da; found: 315.0895 Da.

*(9H-Fluoren-9-yl)(4-(trifluoromethyl)phenyl)methanol* **4i** (49%); white powder; m.p. 149–150 °C. ^1^H-NMR (200 MHz) (CDCl_3_) 7.72 (d, *J =* 7.6 Hz, 2H); 7.56 (d, *J =* 8.3 Hz, 2H); 7.42–7.21 (m, 8H); 5.31 (d, *J =* 5.3 Hz, 1H); 4.43 (d, *J =* 5.3 Hz, 1H). ^13^C-NMR (50 MHz) (CDCl_3_) 145.7 (CIV); 142.8 (CIV); 141.6 (2CIV); 141.8 (2CIV); 127.8 (2CHar); 127.0 (2CHar); 126.9 (CHar); 126.7 (CHar); 125.8 (CHar); 125.3 (CHar); 125.0 (CHar); 124.9 (CHar); 124.8 (CIV); 120.0 (CHar); 119.9 (CHar); 75.6 (CH); 54.6 (CH). HRMS (ESI^+^): *m/z* [M + Na]^+^ calculated for C_21_H_15_OF_3_Na^+^: 363.0967 Da; found: 363.0969 Da.

*3′-(4-(Trifluoromethyl)phenyl)spiro[fluorene-9,2′-oxirane]* **5i** (6%); yellow powder; m.p. 75–76 °C. ^1^H-NMR (200 MHz) (CDCl_3_) 7.67–7.16 (m, 10H); 6.90–6.63 (m, 1H); 6.39–6.35 (dd, *J =* 6.4 et 1.4 Hz, 1H); 4.87 (s, 1H). ^13^C-NMR (50 MHz) (CDCl_3_) 145.3 (CIV); 144.2 (CIV); 141.3 (CIV); 140.2 (CIV); 140.0 (CIV); 129.7 (CHar); 129.4 (CHar); 127.6 (CIV); 127.5 (2CHar); 127.4 (2CHar); 125.5 (CHar); 124.1 (CHar); 123.7 (CHar); 123.6 (CHar); 120.1 (CHar); 120.0 (CIV); 84.7 (CIV); 78.9 (CH). HRMS (ESI^−^): *m/z* [M − H]^−^ calculated for C_21_H_12_OF_3_^−^: 337.0846 Da; found: 337.0846 Da.

*(9H-Fluoren-9-yl)(furan-2-yl)methanol* **4j** (28%); brown powder; m.p. 91–92 °C. ^1^H-NMR (200 MHz) (CDCl_3_) 7.75 (d, *J =* 7.7 Hz, 2H); 7.50–7.20 (m, 6H); 7.05 (d, *J =* 7.6 Hz, 1H); 6.40–6.39 (m, 1H); 6.15–6.14 (m, 1H); 5.03 (d, *J =* 6.2 Hz, 1H); 4.56 (d, *J =* 6.2 Hz, 1H). ^13^C-NMR (50 MHz) (CDCl_3_) 154.9 (CIV); 143.1 (CIV); 143.0 (CIV); 141.7 (2CIV); 127.7 (2CHar); 127.0 (2CHar); 126.0 (2CHar); 124.9 (CHar); 119.7 (2CHar); 110.5 (CHar); 107.4 (CHar); 70.3 (CH); 52.4 (CH). HRMS (ESI^+^): *m/z* [M + Na]^+^ calculated for C_18_H_14_O_2_Na^+^: 285.0886 Da; found: 285.0884 Da.

*3′-(Furan-2-yl)spiro(fluorene-9,2′-oxirane)* **5j** (19%); brown crystals; m.p. 135–136 °C. ^1^H-NMR (200 MHz) (CDCl_3_) 8.70–8.63 (m, 2H); 8.45–8.41 (m, 1H); 7.83–7.50 (m, 7H); 6.78–6.70 (m, 2H). ^13^C-NMR (50 MHz) (CDCl_3_) 148.7 (CIV); 148.3 (CIV); 143.4 (CHar); 131.6 (CIV); 131.4 (CIV); 128.0 (CHar); 127.1 (CHar); 126.7 (CHar); 126.4 (CIV); 124.9 (CHar); 124.8 (CHar); 124.3 (CHar); 123.5 (CHar); 122.7 (CHar); 122.5 (CHar); 111.8 (CHar); 111.4 (CHar); 106.5 (CIV).

*(9H-Fluoren-9-yl)(pyridin-4-yl)methanol*: **4k** (42%); orange powder; m.p. 235–236 °C; ^1^H-NMR (200 MHz) (DMSO-*d_6_*) 8.08 (d, *J =* 6.0 Hz, 2H); 7.96–7.92 (m, 2H); 7.87–7.83 (m, 2H); 7.44–7.36 (m, 4H); 6.74 (d, *J =* 4.6 Hz, 1H); 6.68 (d, *J =* 6.0 Hz, 2H); 5.50 (d, *J =* 4.6 Hz, 1H). ^13^C-NMR (50 MHz) (DMSO-*d_6_*) 150.2 (CIV); 147.0 (2CHar); 143.2 (2CIV); 140.8 (2CIV); 127.2 (2CHar); 126.9 (2CHar); 125.8 (2CHar); 122.1 (2CHar); 118.9 (2CHar); 73.0 (CH); 63.73 (CH). HRMS (ESI^+^): *m/z* [M − H]^−^ calculated for C_19_H_14_NO^−^: 272.1081 Da; found: 272.1081 Da.

*4-(Spiro(fluorene-9,2'-oxiran)-3'-yl)pyridine*: **5k** (11%); Yellow powder; m.p. 197–198 °C ^1^H-NMR (200 MHz) (DMSO-*d_6_*) 8.13 (d, *J =* 8.0 Hz, 1H); 7.88 (d, *J =* 7.0 Hz, 2H); 7.36–7.18 (m, 4H); 6.50 (d, *J =* 7.0 Hz, 2H); 6.22 (d, *J =* 5.9 Hz, 1H); 5.83 (d, *J =* 5.9 Hz, 1H). ^13^C-NMR (50 MHz) (DMSO-*d_6_*) 147.7 (2CHar); 144.4 (2CIV); 143.5 (2CIV); 139.0 (CIV); 129.3 (CHar); 129.2 (CHar); 127.5 (CHar); 127.3 (CHar); 126.7 (CHar); 125.1 (CHar); 122.4 (2CHar); 119.8 (CHar); 119.6 (CHar); 76.9 (CH); 75.6 (CH). HRMS (ESI^+^): *m/z* [M + Na]^+^ calculated for C_19_H_13_NONa^+^: 294.0889 Da; found: 294.0891 Da.

*(9H-Fluoren-9-yl)(pyridin-3-yl)methanol* **4l** (19%); white powder; m.p. 253–254 °C. ^1^H-NMR (200 MHz) (DMSO-*d_6_*) 8.16–8.14 (dd, *J =* 4.7 et 1.7 Hz, 1H); 7.88 (d, *J =* 1.9 Hz, 1H); 7.76–7.73 (m, 1H); 7.56–7.45 (m, 3H); 7.34–7.26 (m, 4H); 7.08–7.04 (dt, *J =* 7.9 et 1.9, 1H); 6.95–6.90 (dd, *J =* 7.7 ou 7.9 et 4.7 Hz, 1H); 6.03 (s, OH); 5.83 (d, *J =* 3.7 Hz, 1H); 5.17 (d, *J =* 3.7 Hz, 1H). ^13^C-NMR (50 MHz) (DMSO-*d_6_*) 148.4 (CHar); 147.3 (CHar); 146.3 (2CIV); 139.8 (2CIV); 134.2 (CHar); 128.2 (CHar); 128.1 (CHar); 126.7 (2CHar); 126.6 (CIV); 125.6 (CHar); 124.5 (CHar); 121.4 (CHar); 119.2 (CHar); 119.0 (CHar); 83.71 (CH); 76.3 (CH). HRMS (ESI^+^): *m/z* [M + H]^+^ calculated for C_19_H_16_NO^+^: 274.1226 Da; found: 274.1226 Da.

*Ethyl 2-(9H-fluoren-9-yl)-2-hydroxypropanoate* **4m** (47%): orange oil. ^1^H-NMR (200 MHz) (CDCl_3_) 7.73 (d, *J =* 7.3 Hz, 2H); 7.63–7.54 (m, 2H); 7.39–7.35 (m, 4H); 4.31 (s, 1H); 4.05 (q, *J =* 7.1 Hz, 2H); 1.60 (s, 3H); 1.04 (t, *J =* 7.1 Hz, 3H). ^13^C-NMR (50 MHz) (CDCl_3_) 175.3 (CIV); 143.0 (CIV); 142.6 (CIV); 142.2 (CIV); 141.8 (CIV); 127.9 (CHar); 127.7 (CHar); 126.7 (2CHar); 126.0 (CHar); 125.7 (CHar); 119.7 (CHar); 119.6 (CHar); 77.2 (CIV); 61.7 (CH_2_); 56.0 (CH); 23.9 (CH_3_); 13.7 (CH_3_). HRMS (ESI^+^): *m/z* [M + NH_4_]^+^ calculated for C_18_H_22_NO_3_^+^: 300.1594 Da; found: 300.1592 Da.

*Methyl 2-(9H-fluoren-9-yl)-3,3,3-trifluoro-2-hydroxypropanoate* **4n** (41%); yellow powder; m.p. 94–95 °C. ^1^H-NMR (200 MHz) (CDCl_3_) 7.77–7.71 (m, 3H); 7.45–7.39 (m, 2H); 7.33–7.25 (m, 3H); 4.61 (s, 1H); 3.70 (s, 3H). ^13^C-NMR (50 MHz) (CDCl_3_) 169.2 (CIV); 142.7 (CIV); 142.1 (CIV); 140.1 (CIV); 139.8 (CIV); 128.5 (CHar); 128.3 (CHar); 126.7 (CHar); 126.8 (2CHar); 125.5 (CHar); 119.8 (CHar); 119.6 (CHar); 80.0 (CIV); 79.5 (CIV); 53.8 (CH); 50.2 (CH_3_). HRMS (ESI^+^): *m/z* [M + NH_4_]^+^ calculated for C_17_H_17_NO_3_F_3_^+^: 340.1155 Da; found: 340.1157 Da.

*Ethyl 2-(9H-fluoren-9-yl)-2-hydroxyacetate* **4o** (25%): yellow powder; m.p. 135–136 °C. ^1^H-NMR (200 MHz) (CDCl_3_) 7.75 (d, *J =* 7.1 Hz, 2H); 7.65 (m, 1H); 7.43–7.26 (m; 5H); 4.94 (d, *J =* 2.7 Hz, 1H); 4.43 (d, *J =* 2.7 Hz, 1H); 4.08 (q, *J =* 7.1 Hz, 2H); 1.03 (t, *J =* 7.1 Hz, 3H). ^13^C-NMR (50 MHz) (CDCl_3_) 173.2 (CIV); 143.3 (2CIV); 142.2 (2CIV); 127.8 (CHar); 127.7 (CHar); 127.2 (CHar); 127.0 (CHar); 124.7 (CHar); 124.5 (CHar); 119.9 (CIV); 119.8 (CHar); 72.6 (CH); 61.6 (CH); 48.31 (CH_2_); 13.7 (CH_3_). HRMS (ESI^+^): *m/z* [M + H]^+^ calculated for C_17_H_17_O_3_^+^: 269.1172 Da; found: 269.1171 Da.

*Diethyl 2-(9H-fluoren-9-yl)-2-hydroxymalonate* **4p** (70%): white powder; m.p. 75–76 °C. ^1^H-NMR (200 MHz) (CDCl_3_) 7.74 (d, *J =* 7.4 Hz, 2H); 7.49 (d, *J =* 7.4 Hz, 2H); 7.43–7.36 (m, 2H); 7.30–7.22 (m, 2H); 4.93 (s, 1H); 4.25 (q, *J =* 7.2 Hz, 4H); 3.88 (bs, 1H); 1.19 (t, *J =* 7.2 Hz, 6H). ^13^C-NMR (50 MHz) (CDCl_3_) 169.5 (2CIV); 142.1 (2CIV); 141.6 (2CIV); 128.0 (2CHar); 127.0 (2CHar); 125.3 (2CHar); 119.7 (2CHar); 81.3 (CIV); 62.6 (2CH_2_); 54.6 (CH); 13.7 (2CH_3_). HRMS (ESI^–^): *m/z* [M + H]^+^ calculated for C_20_H_21_O_5_^+^: 341.1384 Da; found: 340.1311 Da.

### 3.4. Reactivity of 9-Bromofluorene and 4-Cyanobenzaldehyde in the Presence of Triethylamine

Triethylamine (150 mg, 1.2 mmol) was added to a stirred solution of fluorenyl bromide **1** (100 mg, 0.5 mmol) with the 4-cyanobenzaldehyde (160 mg, 1.2 mmol) and a spatula of sodium sulfate in 4 mL of DMF, under air. The mixture was then stirred at −20 °C for 1 h and warmed to room temperature over a period of 24 h. Then 0.5 mL of water was added to quench the reaction. The solution was extracted with dichloromethane (3 × 30 mL), the combined organic layers were washed with brine (3 × 40 mL), and dried over MgSO_4_. The crude product was then obtained after evaporation of the solvent under reduced pressure. Purification by silica gel chromatographic column (dichloromethane: methanol) gave the 20% of the unreacted 9-bromofluorene with 15% yield of the 4-((9*H*-fluoren-9-ylidene)methyl)benzo-nitrile **8c** and 7% yield of the 4-(spiro(fluorene-9,2′-oxiran)-3′-yl)benzonitrile **5f** gave the unreacted 9-bromofluorene (20%) with 4-((9*H*-fluoren-9-ylidene)methyl)benzonitrile **6f** (15%) and 4-(spiro(fluorene-9,2′-oxiran)-3′-yl)benzonitrile **5f** (7%).

*4-((9H-Fluoren-9-ylidene)methyl)benzonitrile*: **6f** (15%) yellow powder; m.p. 149–150 °C (Lit.: 150–151). ^1^H-NMR (200 MHz) (CDCl_3_) 7.97–7.94 (m, 1H); 7.75–7.73 (m, 1H); 7.41–7.30 (m, 6H); 7.12 (d, *J =* 8.3 Hz, 2H); 7.12 (d, *J =* 8.3 Hz, 2H); 6.80 (bs, 1H). Physical and spectroscopic data agree with those reported in the literature [38].

## 4. Conclusions

This strategy afforded a novel and convenient synthesis of various 9-fluorenylidene derivatives under mild reaction conditions. TDAE was used to generate the fluorenyl anion in situ and under practical conditions, which could be extended to electrophiles. In this study, TDAE, or another base present in the reaction mixture, was also able to promote the formation of epoxide derivatives. Moreover, because of their structural analogy with recently reported compounds, the anti-nociceptive properties of all intermediates are under active investigation.

## Figures and Tables

**Table 1:** Reactions of fluorenyl bromide **1** and aldehyde **2a** with TDAE under various conditions.

Entry	Aldehyde 2a (eq.)	TDAE (eq.)	Solvent	Yield ^1,2^ of Dimer 3	Yield ^1,2^ of 4a
1	2	1	DMF	44%	29%
2	3	1	DMF	8%	50%
3	3	2	DMF	10%	48%
4	4	1	DMF	8%	46%
5	3	1	THF	12%	48%
6	3	1	DMF ^3^	9%	56%
7	3	1	CH_3_CN	16%	30%
8	3	1	DMSO	20%	12%
9	3	1	pyridine	11%	34%

^1^ Isolated yields; ^2^ Typical procedure: to a vigorously stirred solution of fluorenyl bromide **1** derivative in 4 mL anhydrous solvent, at −20 °C, under N_2_; ^3^ Moist DMF was used in the presence of a spatula of sodium sulfate in the reaction mixture and the reaction was run under air.

**Table 2:**
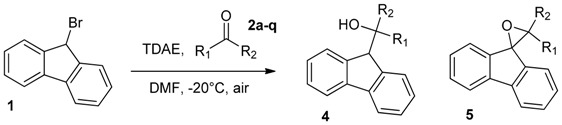
The scope of the reactivity of **1** in the presence of various electrophiles under TDAE reaction conditions.

Entry	Electrophiles			Yield ^1,2^ of 4	Yield ^1,2^ of 5
		R_1_	R_2_		
1	**2a**	4-ClC_6_H_4_	H	56% (**4a**)	14% (**5a**)
2	**2b**	C_6_H_5_	H	57% (**4b**)	17% (**5b**)
3	**2c**	4-BrC_6_H_4_	H	75% (**4c**)	10% (**5c**)
4	**2d**	4-OMeC_6_H_4_	H	34% (**4d**)	
5	**2e**	4-FC_6_H_4_	H	54% (**4e**)	
6	**2f**	4-CNC_6_H_4_	H	41% (**4f**)	30% (**5f**)
7	**2g**	4-MeC_6_H_4_	H	24% (**4g**)	
8	**2h**	4-(NO_2_)C_6_H_4_	H		17% (**5h**)
9	**2i**	4-CF_3_ C_6_H_4_	H	49% (**4i**)	6% (**5i**)
10	**2j**	2-furyl	H	28% (**4j**)	19% (**5j**)
11	**2k**	4-pyridinyl	H	42% (**4k**)	11% (**5k**)
12	**2l**	3-pyridinyl	H	19% (**4l**)	
13	**2m**	Me	CO_2_Et	47% (**4m**)	
14	**2n**	CF_3_	CO_2_Me	41% (**4n**)	
15	**2o**	H	CO_2_Et	25% (**4o**)	
16	**2p**	CO_2_Et	CO_2_Et	70% (**4p**)	
17	**2q**	C_6_H_5_	Et		

^1^ Isolated yields; ^2^ Typical procedure: to a vigorously-stirred solution of 9-bromofluorene **1** in 4 mL moist DMF with a spatula of sodium sulfate, under air, TDAE was slowly added at −20 °C. The solution was then stirred 1 h at this temperature followed by 2 h at ambient temperature.
